# Effect of Cold Plasma on Meat Cholesterol and Lipid Oxidation

**DOI:** 10.3390/foods9121786

**Published:** 2020-12-01

**Authors:** Juan M. Pérez-Andrés, Janna Cropotova, Sabine M. Harrison, Nigel P. Brunton, Patrick J. Cullen, Turid Rustad, Brijesh K. Tiwari

**Affiliations:** 1 Food Chemistry and Technology, Teagasc Food Research Centre, 15 Dublin, Ireland; juan.perezandres@teagasc.ie; 2 BioPlasma Research Group, School of Food Science and Environmental Health, Technological University Dublin, Cathal Brugha Street, 1 Dublin, Ireland; patrick.cullen@sydney.edu.au; 3 Norwegian University of Science and Technology, Department of Biotechnology and Food Science, 7034 Trondheim, Norway; janna.cropotova@ntnu.no (J.C.); turid.rustad@ntnu.no (T.R.); 4 UCD School of Agriculture & Food Science, University College Dublin, 4 Dublin, Ireland; sabine.harrison@ucd.ie (S.M.H.); nigel.brunton@ucd.ie (N.P.B.); 5 School of Chemical and Biomolecular Engineering, University of Sydney, Darlington, NSW 2008, Australia

**Keywords:** cholesterol, lipids, cold atmospheric plasma, oxidation

## Abstract

Cold atmospheric plasma (CAP) is a novel non-thermal technology with potential applications in inactivating microorganisms in food products. However, its impact on food quality is not yet fully understood. The aim of this research is to study the impact of in-package plasma technology on the stability of cholesterol and total lipid in four different types of meat (beef, pork, lamb and chicken breast). Additionally, any changes in the primary or secondary lipid oxidation, which is undesirable from a health perspective, is investigated. CAP was not found to have any impact on the cholesterol or lipid content. However, higher peroxide and thiobarbituric acid reactive substances (TBARS) values were found for the treated samples, indicating that plasma can induce the acceleration of primary and secondary lipid oxidation. Finally, color was not affected by the treatment supporting the suitability of the technology for meat products.

## 1. Introduction

Globally, meat consumption continues to increase. According to the latest report from the Food and Agriculture Organization [1], the production of meat has increased by 1.7% from 2017 to 2018, reaching a value of 335 million tons (36.9% poultry, 36.6% pig, 21.9% bovine and 4.6% ovine). Cholesterol is present in all these meat products and plays an important role as a structural component of the phospholipid bilayer of the plasma membrane of eukaryotic cells. Cholesterol is fitted into membrane bilayers with its long axis, preventing the crystallization of fatty acyl chains and thereby modifying the activity of membrane-bound enzymes [2]. It also has vital functions in the metabolism and function of body tissue [3]. Cholesterol is an essential precursor for the synthesis of vitamin D, bile, bile acids salts, steroids and hormones [4]. For instance, vitamin D3 is a derivative of cholesterol and is formed in the skin from 7-dehydrocholesterol. Moreover, deficiencies in cholesterol during embryogenesis and organogenesis can cause severe abnormalities in the fetus [5].

In contrast, high concentrations of cholesterol in cells can be cytotoxic and pro-inflammatory [3]. Furthermore, high levels in cholesterol can lead to the development of diseases through atherogenesis, the agglomeration of low-density lipoprotein (LDP) cholesterol on the arterial wall, creating plaques which can obstruct blood flow leading to cardiovascular diseases [6]. In addition, a disturbance on the cholesterol metabolism can also cause numerous chronic diseases including cancer, as well as disorders of metabolic and neurological tissues [6]. However, some authors suggest that only oxidized cholesterol can contribute to the pathophysiology of human diseases such as carcinogenic, cytotoxic, mutagenic, atherogenic and neurodegenerative diseases [2]. The formation of cholesterol oxidation products in food are initiated by free radicals, which can be generated by auto-oxidation, photo-oxidation and thermo-oxidation [5], leading to a chain reaction mechanism [7].

**Figure 1:**
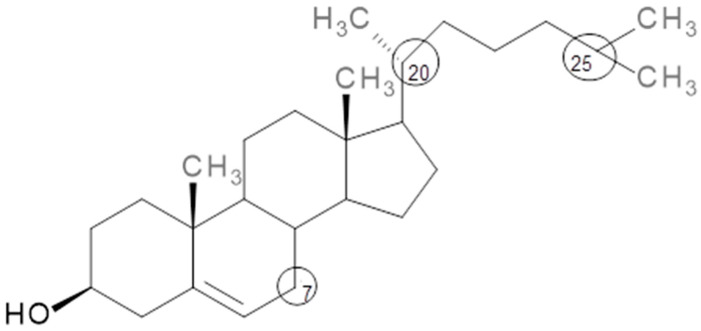
Cholesterol structure. Circles indicate the most reactive carbon.

Cholesterol (5*α*-cholesten-3*β*-ol) is a lipid which belongs to the family of sterols (Figure 1). It is characterized by its reactive behavior, especially on C7, C20 and C25, leading to the formation of oxysterols which have similar structures but with the addition of other functional groups such as hydroxyl, hydroperoxide, ketone and epoxide [8,9]. The most common oxidation products from C7 are 7-*α*-hydroxycholesterol, 7-ketocholesterol and 7-*β*-hydroxycholesterol, while 20-hydroxycholesterol and 25-hydroxicholesterol are formed when the oxidation occurs on C20 and C25, respectively [8]. These compounds belong to the group of oxysterols, a class of compounds reported to be involved in several neurodegenerative diseases including Huntington’s, Parkinson’s and Alzheimer’s disease [10].

Cold atmospheric plasma (CAP) is a novel technology with several uses in various industries. A plasma atmosphere is made up of different free radical molecules, electrons, UV-photons, positive and negative ions, ozone as well as carbon and nitrogen oxides, which vary according to the gas applied [11]. Recently, the technology has been proposed as a decontamination step for the preservation and safety assurance of foods [12,13,14]. Wang et al. [15] described a dielectric barrier discharge operating at 80 kV for 180 s which was applied to chicken breasts packaged in food trays in both atmospheric air and modified atmosphere gas (65% O_2_, 30% CO_2_ and 5% N_2_). While no significant reduction of microbial populations was found for samples packaged using atmospheric air, they reported that the treatment was effective for chicken packaged under modified atmosphere, suggesting that CAP treatment could increase shelf-life from 7 days to at least 14 days. Furthermore, Yong et al. [12] treated beef jerky at 15 kV for 2.5, 5, and 10 min using a dielectric barrier discharge system which resulted in a reduction of *Listeria monocytogenes*, *Escherichia coli*, *Salmonella typhimurium* and *Aspergillus flavus* for all treatment times. Significant reductions of *Listeria monocytogenes* and *Escherichia coli* were also found for pork loin treated by a dielectric barrier discharge (DBD) plasma operating at 3 kV with a 30 kHz bipolar square wave for treatment times of 5 and 10 min [13].

However, it is shown that these radical species present in the plasma may modify food components, leading to their oxidation [14]. Lipid oxidation has long been considered a radical chain reaction triggered by hydrogen abstractions with hydrogen peroxides being considered the first stable products. However, following their decomposition, hydrogen peroxides may generate secondary lipid oxidation products [16]. When investigating lipid oxidation, simultaneous pathways for generation of secondary lipid oxidation products should be considered; various reactions of addition, rearrangement or dismutation of lipid peroxyl radicals (LOO) can lead to further formation of dimers, epoxides, aldehydes or ketones in parallel to hydroperoxides. Therefore, to fully assess lipid oxidation along with the determination of lipid peroxides, it is important to analyze a complex mix of secondary lipid oxidation products [16,17]. The thiobarbituric acid reactive substances (TBARS) assay is a commonly used method to assess secondary lipid oxidation by measuring the content of a secondary degradation product, namely malonaldehyde. While heated in an acidic medium, malonaldehyde reacts with thiobarbituric acid (TBA) to form a pink Schiff base adduct with an absorption maximum in the region of 532–535 nm [18]. However, the TBARS method also determines a complex mixture of various other secondary oxidation products including alkanals, alkenals, alkadienals and others which react with TBA. Nevertheless, it is widely used as an indicator of lipid oxidation, particularly in meat products [16].

Using TBARS to detect lipid oxidation, it has been reported that cold atmospheric plasma can accelerate the production of peroxides, as well as lipid and protein oxidation in pork during storage [19]. A flexible thin-layer dielectric barrier discharge plasma also oxidized lipids in beef jerky [12], as well as pork butt and beef loin [20]. In another study, it was reported that TBARS values were significantly higher in dry-cured beef, “bresaola” after plasma treatment [14]. Furthermore, Kim et al. [21] also found an increase in lipid oxidation products in bacon after cold atmospheric plasma treatment by deploying the TBARS assay. Finally, CAP not only had an impact on the quality of meat products; it also accelerated the oxidation of proteins in mackerel fillets [22].

There is a need for more research on the effects of plasma on meat chemistry before adoption of this technology by the industry. It is important to evaluate how plasma may affect all the components present in the matrix, and to determine if CAP could increase the shelf-life of the products without affecting their quality or safety or exposing the consumers to any health risk.

Hence, the objective of this study was to investigate if CAP could cause any undesirable effects on lipids such as the oxidation or degradation of cholesterol leading to the formation of oxidized compounds. To investigate the effects, we first applied CAP directly to a cholesterol standard. The results were then compared to the effects observed for four different meat minces, namely beef, lamb, pork and chicken, where the complexity of the matrix may display protective properties. In addition, total lipid oxidation as well as possibly changes in color of the meats were investigated following CAP treatment.

## 2. Materials and Methods 

### 2.1. Chemicals and Reagents

Cholesterol standard (purity 98%), the internal standard 5*α*-cholestan-3*β*-ol (purity 98%), pyridine, bis(trimethylsilyl) trifluoroacetamide (BSTFA) with 1% of trimethylchlorosilane (TMCS) and potassium hydroxide were purchased from Sigma-Aldrich (Arklow, Co., Wicklow, Ireland). Chloroform was purchased from Sigma-Aldrich (Oslo, Norway). Methanol, hexane and dichloromethane were purchased from Fisher Scientific (Dublin, Ireland). Ultra-pure water (18.2 MΩ cm^−1^) was generated in-house using a Millipore water purification system (Millipore, Cork, Ireland). All chemicals were GC grade.

### 2.2. Cholesterol Standard Preparation

A 1 mg/mL solution of cholesterol standard in methanol was prepared. An aliquot of 200 µL of this solution was dispensed into a glass petri dish (2.5 cm radius × 1.5 cm height), and the solvent was let to evaporate. Then, the petri dish was sealed using Parafilm.

### 2.3. Cholesterol Standard Plasma Treatment

**Figure 2:**
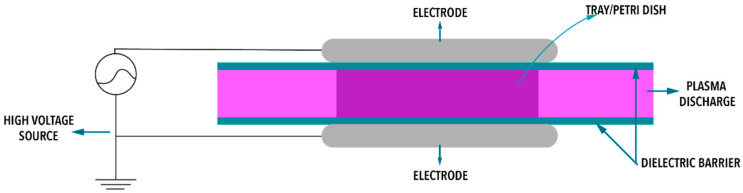
Schematic of the cold atmospheric plasma (CAP) setup.

A petri dish containing cholesterol standard as previously described was treated using an in-house dielectric barrier discharge atmospheric plasma system, which was described previously [23]. The atmosphere used to fill the petri dish in all our experiments was standard laboratory air. This petri dish was placed between two circular aluminum electrodes (outer diameter = 158 mm) separated by two polypropylene (PP) dielectric layers (2 mm thickness) as per Figure 2. The distance between the dielectric layers was the height of the petri dish (1.5 cm). Two different voltages, 60 kV and 80 kV RMS (root mean square) were applied for two different durations, 5 and 10 min, in triplicate; these conditions were previously shown to control microbial growth in mackerel [24]. After the treatment, control (*n* = 3) and treated samples (*n* = 3) were kept at 4 °C for 24 h to ensure the induced plasma components can interact with the cholesterol as suggested by Ziuzina et al. [25]. Following this storage, 200 µL of the internal standard (5*α*-cholestan-3*β*-ol; 50 mg/mL in dichloromethane) was added to the petri dish and left to stand until full evaporation of the dichloromethane. Once the dichloromethane was evaporated, the residues of both, the cholesterol and internal, standards were reconstituted in 10 mL of dichloromethane and kept at −80 °C until the day of analysis.

### 2.4. Meat Sample Preparation

Fresh meat minces from four different species (beef, lamb, pork and chicken breast) were purchased at a local butcher in Dublin in November 2018. Neither the type of muscle used to produce the mince, nor the origin of the meat were identified at this stage. Using a spoon, a homogenous portion of 100 g of each was added to a black amorphous polyethylene terephthalate (APET/PE) tray (195 × 155 × 30 mm), sealed under atmospheric air conditions using a low oxygen permeable barrier polyvinyl-chloride film (3 cm^3^/m^2^/24 h at Standard Temperature and Pressure (STP); Versatile Packaging, Silverstream, Co. Monaghan, Ireland), and packaged individually (Ilpra Foodpack VG 400 Packaging Machine, Mortara, Italy) to mirror common commercial practice. Samples of each meat were divided in four different batches: control and treated with 24 h of storage post-treatment as well as control and treated with 7 days of storage post-treatment. All conditions were prepared in triplicate.

### 2.5. Meat Sample Plasma Treatment

The same dielectric barrier discharge system as described in Section 2.3 was used for this study. The samples were treated with an in-package mode, where the plasma was induced inside the gas contained inside the sealed package. Each packed sample was placed between the two electrodes separated by 3 cm, i.e., the height of the tray. Ten minutes of treatment was performed at a discharge voltage of 80 kV RMS; these settings have previously been shown to control microbial growth in mackerel [24]. Similar to the cholesterol standard study, once the CAP treatment was finished both the control and plasma samples were kept at 4 °C for either 24 h or 7 days. Following this storage, i.e., either 24 h or 7 days, all samples were individually vacuum-packed and stored at −80 °C until the day of the analysis. All conditions were prepared in triplicate.

### 2.6. Cholesterol Analysis

#### 2.6.1. Cholesterol Extraction

Cholesterol was quantified according to [26]. This method has previously been fully validated for the determination of cholesterol in turkey meat products. Briefly, approximately 0.5 g of chilled meat mince sample, 200 µL of internal standard (5*α*-cholestan-3*β*-ol, concentration: 50 mg/mL) and 30 mL of 4 M KOH in methanol were mixed and homogenized using an Ultraturrax homogenizer (Labortechnik, Staufen, Germany) at 13,500 rpm for 30 s in a 50 mL tube. Saponification was carried out by placing the sample into a water bath at 60 °C for one hour. After cooling down, a liquid-liquid extraction was performed by adding 5 mL of Milli Q water and 5 mL of heptane; phase separation was achieved by centrifuging the sample at 4000 rpm (2500× *g*) for 10 min. The organic phase was transferred to another tube and the extraction was repeated twice more by adding 5 mL of heptane each time. All organic layers were combined and dried using a sample concentrator and reconstituted into 10 mL of dichloromethane. Derivatization of cholesterol was performed by mixing 0.5 mL of this extract, 200 µL of BSTFA-TMCS, 100 µL of pyridine and leaving them in a water bath for 15 min at 70 °C. After cooling down, the solution was diluted to 10 mL with dichloromethane and an aliquot was transfer to the vial to be injected into the gas chromatography system.

#### 2.6.2. Gas Chromatography-Flame Ionization Detector Analysis

Separation was carried out using a Clarus 580 Gas Chromatograph (Perkin Elmer, Waltham, MA, USA) fitted with a flame ionization detector (GC-FID) set at 260 °C according to [26]. These authors previously showed the method to have a limit of quantitation of 0.1 µg/mL (equivalent to 0.4 mg cholesterol per g meat); a limit of detection was not reported. The separation and quantification of cholesterol was carried out employing a ZB-5 capillary column (Phenomenex, Torrance, CA, USA) with a film thickness of 0.25 µm and a length of 30 m × 0.25 mm. The injection volume was 0.5 µL and the inlet temperature was set to 200 °C. Hydrogen was flushed at a constant flow of 2.0 mL/min, and the split ratio was set at 5:1. The oven temperature started at 180 °C with an initial temperature ramp of 8.0 °C/min to 260 °C followed be a second temperature ramp of 2 °C/min to 280 °C which was held for 10 min.

The content of cholesterol was calculated using following equation (Equation (1)) according to Grasso et al. [26].

(1) $$\mathrm{Content}\;(\mathrm{mg}/g)\;=\;\times \frac{\mathrm{Weight}\;\mathrm{ISTD}}{\mathrm{Weight}\;\mathrm{Sample}}\times \;\frac{\mathrm{IS}\;\mathrm{Purity}}{\mathrm{RRF}}\times \;20$$

where, IS Purity is the purity of the internal standard as given on the certificate of analysis, RRF is the relative response factor for cholesterol (namely 1.001), and 20 is the dilution factor.

### 2.7. Lipid Content

The total lipid content was determined using the Bligh & Dyer (B & D) method which applies a mixture of chloroform, methanol and Milli Q water for the extraction of lipids from the muscle tissue [27]. The extraction was performed in duplicate. Briefly, experimental meat samples were minced with a kitchen blender (Bosch MSM87140, Frankfurt, Germany) and 10 g of the obtained mince was transferred into centrifuge tubes. The centrifuge tubes were kept on ice during the whole procedure. Then, distilled water (10 mL), chloroform (20 mL) and cold methanol (40 mL) were added to each tube. The mixture was homogenized using an Ultraturrax (IKA T18, Staufen, Germany) for 2 min at 9000 rpm. Additional amount of chloroform (20 mL) and distilled water (20 mL) was added separately, and the mixture was homogenized again using the Ultraturrax for 30 s after each addition. After the homogenization, the tubes were centrifuged (Hettich Universal 16A Centrifuge, Berlin, Germany) for 10 min at 11,800 rpm. An aliquot of the chloroform phase (2 mL) was collected from the bottom of each of the tubes and transferred into a pre-weighed Kimax glass tube (10 mL). The Kimax glass tube with chloroform phase was placed in an evaporation unit and kept at 60 °C with N_2_-stream for 1 h. After the evaporation, the tubes were corked, cooled down to room temperature and weighed again. The results are expressed as total lipid (average ± standard deviation) in percentage of wet weight meat sample [27].

The remaining chloroform phase in the centrifuge tubes was collected and transferred to plastic tubes resistant to chloroform, flushed with N_2_-gas and stored at −80 °C prior to analysis of peroxide values (PV) and TBARS.

### 2.8. Peroxide Value

PV was determined by using the iodometric titration method described by [28]. The end point of titration was assessed potentiometrically with an automatic titrator (TitroLine 7800, Xylem Analytics, Mainz, Germany) fitted with a platinum electrode (Pt 62). In line with the AOCS method, the analysis was performed in duplicate and the results were expressed in meq active oxygen/kg lipids as an average ± SD.

### 2.9. TBARS

The TBARS assay assesses secondary lipid oxidation products through a reaction between malondialdehyde and thiobarbituric acid (TBA). It is a good indicator of the general oxidative status in fish products because TBA reacts with a wide range of aldehydes and oxidized molecules derived from lipids and proteins. In complex food matrices such as meat and fish, lipid oxidation may take place via complex pathways due to co-oxidation reactions between lipid radicals, secondary oxidation products, pro-oxidants such as transition metals, blood or myoglobin, as well as other system components such as proteins [17]. Co-oxidation reactions result in the oxidation of other food molecules such as proteins with involvement of lipid oxidation intermediates and products [16,18]. Therefore, it is very important to apply a method such as the TBARS assay that can assess the general status of secondary lipid oxidation.

For this study, secondary lipid oxidation was investigated using TBARS determined in the chloroform phase according to the method of Ke and Woyewoda [29]. 1,1,3,3-tetraethoxypropane (T 9889) was used as a standard. The analysis was performed in triplicate and the results were expressed as average ± standard deviation in μMol TBARS/g lipid.

### 2.10. Color

Color characteristics of plasma-treated and untreated meat samples were measured instrumentally using a Minolta Chroma meter CR-400 (Konica-Minolta, Osaka, Japan). For this, meat samples with a thickness of 1 cm were prepared. Before starting the analysis, the instrument was calibrated with a standard white plate. The data was recorded in color coordinates of L* (lightness, black = 0, white = 100), a* (redness > 0, greenness < 0), and b* (yellowness > 0, blueness < 0) according to the Commission Internationale de l’Éclairage (CIE) Lab scale [30]. Three measurements were performed on each of the meat samples, and the average with standard deviation was determined.

### 2.11. Statistical Analysis

Analysis of variance (ANOVA) of dependent variables was carried out using Minitab 17.1.0 (Minitab Inc, State College, PA, USA). Statistics were calculated using a general lineal model (GLM) considering the entire variable as fixed factors. When differences were observed (*p* < 0.05), a Tukey’s multiple comparison was calculated to study the effect of the plasma treatment on the cholesterol. Experiments were performed in triplicate and analyses were carried out in triplicate.

## 3. Results and Discussion

### 3.1. Cholesterol Content

Cholesterol is an important food nutrient due to its role in the biosynthesis of vitamin D, bile acids and steroid hormones such as gonadal (testosterone, estrogens, progesterone) and adrenal (aldosterone, cortisol) [31]. Moreover, it is an important constituent of the cell membrane. For this reason, it is important to study if a novel non-thermal technology such as CAP could have any impact on this micronutrient despite previous research showing that CAP may not be able to significantly penetrate solids [14].

#### 3.1.1. Cholesterol Standard

**Figure 3:**
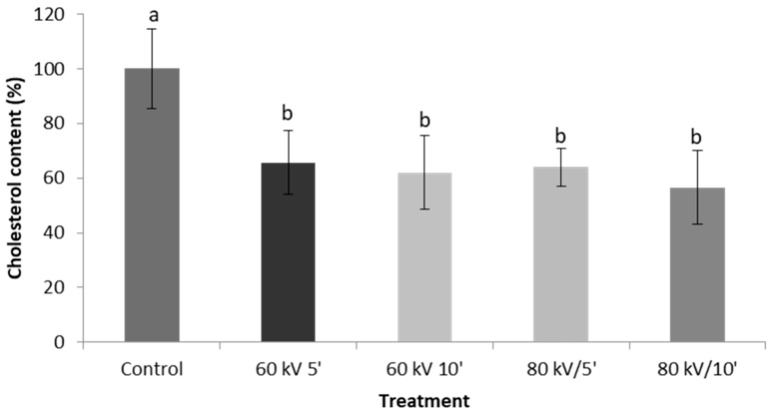
Cholesterol content of control and plasma-treated samples. Different letters indicate a significant difference (*p* < 0.05) for cholesterol at specific treatment conditions.

Cholesterol standard was exposed to CAP at two voltages (60 and 80 kV) for two different durations, namely 5 or 10 min. Overall, results show that CAP has a significant effect (*p* < 0.05) on the cholesterol content of the treated standard with CAP treatment significantly reducing the amount of cholesterol recovered from the petri-dishes. As seen in Figure 3, the cholesterol content decreased by around 35% for all the different treatments, suggesting that this could be because plasma can lead to oxidation and/or degradation of cholesterol. As was mentioned previously, oxidation of cholesterol can be initiated by free radical species, consequently, many of the different radical species which are presented in the plasma bulk could cause this reaction to happen [32]. However, there were no significant differences between the different treatments, i.e., oxidation and/or degradation was not dependent on voltage or duration of treatment.

Although the effect of cold plasma on isolated nutrients has already been reported, to the best of our knowledge, this is the first time that the effect of CAP on cholesterol is reported. However, as the present study used GC-FID for the quantification of cholesterol, it was not possible to identify the breakdown products produced by the plasma. Further research using e.g., a GC coupled to a (tandem) mass spectrometer may give further insight into the processes involved and may help in identifying which carbons in the cholesterol molecule are more prone to oxidation/degradation than others.

Previously, several food protein powders (gelatin, hemoglobin and lung protein extract) were treated at 80 kV (RMS) for 15 min using a dielectric barrier discharge plasma system [33]. Authors observed that the direct treatment of cold plasma on these three protein powders affected their native structure, leading to a significant change in their functional, rheological and gelling properties. Similar results were observed by Ji et al. [34] after treating peanut isolated protein with a different dielectric barrier discharge at 35 kV for 1, 2, 3 or 4 min.

#### 3.1.2. Cholesterol in Meat Products

Since the impact of CAP on fatty acids has been described previously (e.g., [22,24]), cholesterol was chosen as a model to investigate possible oxidation caused by CAP for the present study.

**Figure 4:**
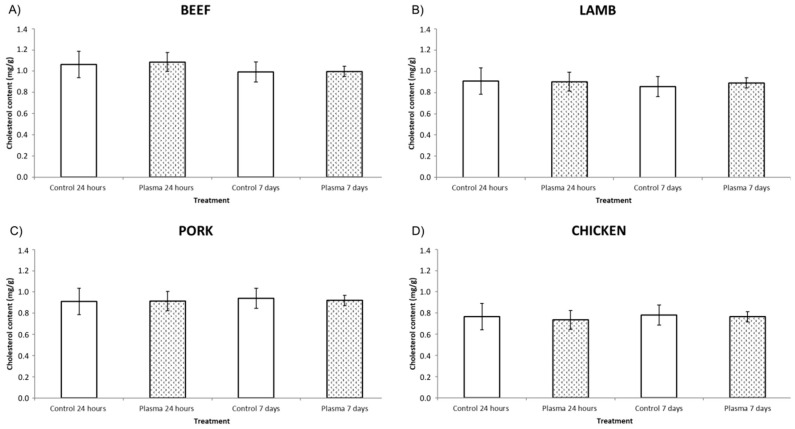
Cholesterol content of control and plasma samples for: (**A**) Beef, (**B**) Lamb, (**C**) Pork, (**D**) Chicken. No significant differences between control and treated sample (*p* > 0.05). All the differences are measured separately for each individual kind of meat. Cholesterol content is given in mg/g meat.

CAP did not have a significant impact on the cholesterol content in any of the four different meat mince samples (*p* > 0.05) as can be seen in Figure 4. This may be because plasma only affects the surface of the meat and hence cannot penetrate into the meat product [14]. On the other hand, the complexity of the matrix and components inside the food could protect the cholesterol from the potential impact of cold plasma. For instance, other lipids present in the meat such as triglycerides in general and polyunsaturated fatty acids more specifically, could be more susceptible to oxidation and/or reaction with the plasma thus protecting cholesterol from interaction with the radical species resulting in cholesterol oxidation and/or degradation. In addition, the presence of naturally occurring antioxidants such as carnosine, anserine, carnitine or taurine, in meat could not only prevent the formation of undesirable oxidation products in general [21] but more specifically the oxidation of cholesterol. Moreover, there are also some antioxidant enzymes (superoxide dismutase, catalase and glutathione peroxidase), vitamins with antioxidant properties (ascorbic acid and α-tocopherol) and minerals like zinc or selenium [35] which could give extra protection against the potential impact of the reactive species present in the plasma atmosphere. Nevertheless, given that the samples were minced, offering a large exposure area, were exposed to extended treatment times and retained in an atmosphere containing induced plasma species for up to 7 days due to the use of the in-package technology, the data indicated no observable effects on the cholesterol contents of the tested meats.

### 3.2. Lipid Content and Oxidation

#### 3.2.1. Lipid Content

**Figure 5:**
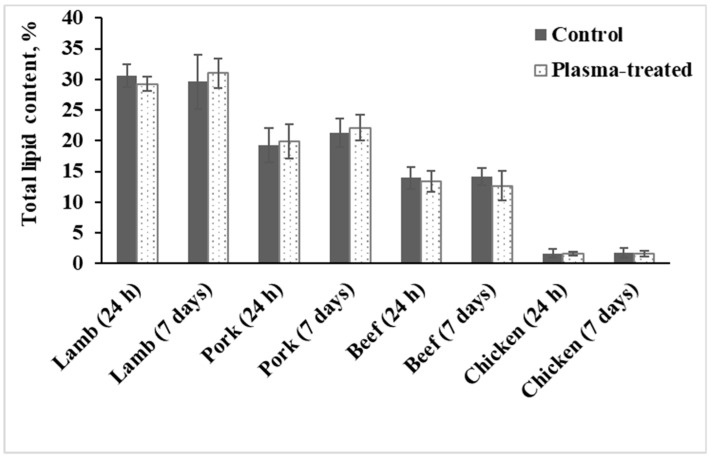
Lipid content in control and plasma-treated meat samples.

Total lipid content in meat samples varied from 1.7 ± 0.7% for chicken (white meat) to 30.6 ± 1.9% for lamb (red meat; Figure 5). However, no significant variation in the total lipid content was found between plasma-treated and untreated samples during the storage period for any of the meats investigated.

#### 3.2.2. Peroxide Value

As the official method used [28] has a detection limit of 0.05 mL lipid per 12 mL chloroform (equivalent to a lipid content of about 5–6%), it was not possible to measure peroxide value in chicken samples due to their very low lipid content (<2% *w*/*w*). However, novel methods such as the one recently published by [36] may give further information on peroxide values of low-lipid samples.

**Figure 6:**
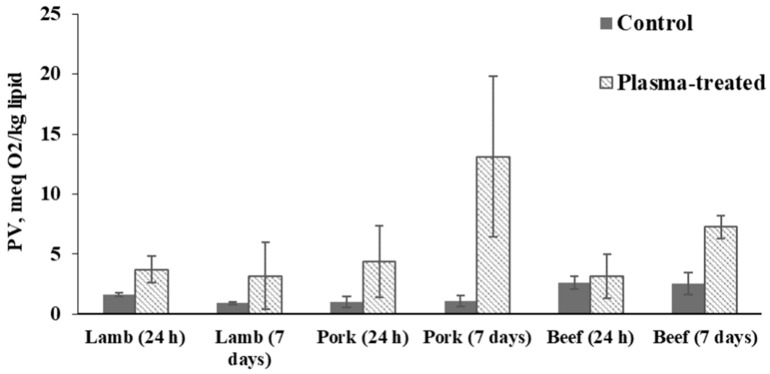
Peroxide value (PV) of control and plasma-treated meat samples.

The rest of the samples with higher lipid content in the tissue, i.e., those with more than 5% lipid content, showed increased peroxide values in all plasma-treated samples compared to non-treated (control) samples (Figure 6). At the same time, only plasma-treated pork and beef samples exceeded the limit for PV established by the CODEX STAN (10 meq O_2_/kg lipid [37]) on day 7 of chilled storage (Figure 5).

Plasma-treatment could result in the accumulation of reactive species accelerating lipid oxidation [38]. This suggestion is supported by the study of Albertos et al. [24] who previously used a non-thermal plasma (NTP) system to treat fresh mackerel fillets. They observed a similar trend for PV results after the treatment of mackerel samples with NTP. The treatment resulted in an over 5-fold increase in peroxide values compared to control samples, with both exposure time and voltage affecting the rate of oxidation [24]. Furthermore, our observations are in line with those made by Yong et al. [12] who reported a significant increase in the PV of beef jerky following flexible thin-layer plasma treatment for 10 min. However, this group only found a significant increase after 10 min of treatment while beef jerky treated for 2.5 or 5 min was not significantly different from the control.

#### 3.2.3. Thiobarbituric Acid Reactive Substances (TBARS)

Lipid oxidation leads to the formation of a very wide range of different oxidation products, making the determination of lipid oxidation challenging. The determination of TBARS is one of the oldest and the most commonly used methods for assessing secondary lipid oxidation status by measuring one of the end product of polyunsaturated fatty acid (PUFA) peroxidation—malondialdehyde [39,40,41,42,43,44,45], but it will also determine other aldehydes [43].

**Figure 7:**
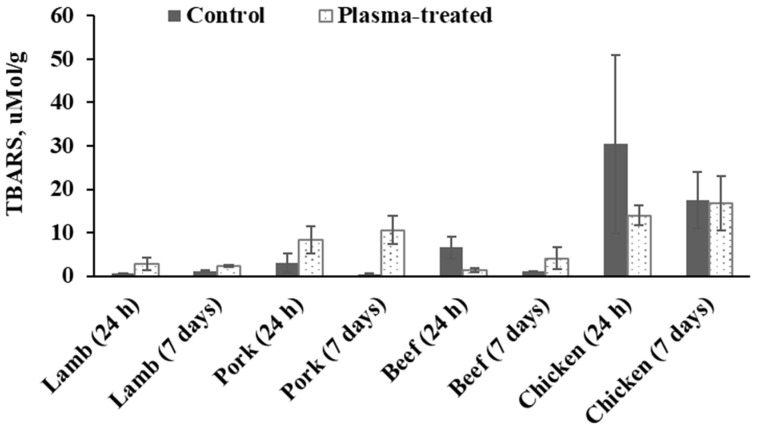
Thiobarbituricacid reactive substances (TBARS) value of control and plasma-treated meat samples. TBARS are presented in µMol/g lipid.

Except for beef samples after 7 days of storage and all the chicken samples, plasma-treated meat samples were characterized with significantly higher TBARS-values compared to the untreated ones (Figure 7). This effect can be explained by the radical-initiating mechanism of plasma treatment as mentioned previously [24]. However, the highest TBARS-values were surprisingly found in chicken samples which contained the lowest lipid content. This may possibly be explained by the typically higher content of PUFAs in chicken as compared to beef or pork (e.g., [46,47]). However, research has also shown that PUFAs vary depending on diet [48]. As the samples for the present study were purchased at a commercial butcher, no information on dietary history or muscle used to produce the mince could be obtained. This information, along with the determination of the fatty acid profile of the samples used in the present study may have assisted in explaining the differences in TBARS found for the different meats.

While the length of this study was chosen to represent shelf-life in a retail setting, experimental duration has recently been extended by several authors in order to investigate long-term impact of plasma treatment on the food matrix. For instance, studies on pork [19] and chicken [49] investigated the impact of plasma for 12 and 21 days, respectively. While [19] reported a significant increase in lipid oxidation on days 4, 8 and 12 for plasma-treated samples as compared to day 0 as well as the control samples, no such observations were made in chicken, even after 21 days of cold storage [49].

Nevertheless, our findings are in contrast to the findings reported by Jayasena et al. [20] who reported a significant increase of TBARS values in beef loin following treatment with flexible thin-layer dielectric barrier discharge plasma for 10 min. Furthermore, these authors only report a significant increase after 10 min treatment while samples treated for 2.5 or 5 min were not significantly different from the control. In addition, the same authors also reported TBARS for pork butt treated for the same durations as the beef loin (i.e., 2.5, 5 and 10 min). However, unlike the beef loin, pork butt samples did not display significant increases in TBARS when compared to the control. In another study [50], ham was packed in three different gas mixtures, namely 20% O_2_, 40% N_2_ and 40% CO_2_ (treatment 1), 50% CO_2_ and 50% N_2_ (treatment 2) or 100% CO_2_ (treatment 3) and treated at 30 kV for either 5 or 10 min using a dielectric barrier discharge (DBD) plasma system. The authors reported that TBARS values were significantly higher for the treated samples compared with the control. A dielectric barrier discharge system was also used to treat chicken breast at 100 kV for 1, 3, and 5 min durations [51]. TBARS for treated samples were significantly higher than for untreated ones. More studies should be performed in this direction to investigate these opposing findings reported thus far.

### 3.3. Color

No significant difference between control and plasma-treated meat samples (for each category of meat) was observed, suggesting that plasma treatment did not have notable effects on the color of the meat tissue. These findings are in line with those reported by Jayasena et al. [20] for beef and pork. This group also reported no significant changes in L* values of beef loin and pork butt while also no significant changes were observed for b* for pork butt. In contrast, however, the same group reported significantly lower a* values for plasma-treated beef loin and pork butt along with significantly higher b* values for beef loin. Finally, Yong et al. [12] reported significantly lower L* values for plasma-treated beef jerky treated for 10 min when compared to the control sample while reporting significantly higher b* values. These authors did not find any significant effect on a* values caused by plasma treatment, in line with the observations made in this study.

## 4. Conclusions

Our results show that the induced reactive species can degrade cholesterol in its pure form. However, the cholesterol content in meat samples was not affected by the treatment, suggesting that the matrix effect plays an important role in protecting this food micronutrient from degradation. In addition, higher peroxide values and TBARS were found for the treated samples as compared to the control samples, indicating that plasma can induce the acceleration of primary and secondary lipid oxidation without affecting absolute lipid content. Finally, color was not affected by the treatment, supporting the suitability of the technology for meat products.

However, further research is required on the impact of this technology on the quality of food products prior to its approval and adoption by regulators and industry, respectively. There is a need to understand the chemical reactions associated with plasma species to avoid quality deterioration. This is particularly true when trying to understand the impact of CAP on lipid fractions and as such, future research should focus on investigating several lipid fractions in parallel such as cholesterol as well as fatty acids to get a clearer understanding of the order in which lipid fractions get oxidized. As our research shows, plasma studies need to be performed on real food products and not only model solutions in order to determine which operational conditions suit the vast array of potential food products that could be treated. Optimization of plasma control parameters, in particular the inducer gas employed, need to be investigated in depth to identify the conditions which can provide the required product safety and yet retain key quality attributes.
